# Maternal and Neonatal Oral Microbiome Developmental Patterns and Correlated Factors: A Systematic Review—Does the Apple Fall Close to the Tree?

**DOI:** 10.3390/ijerph18115569

**Published:** 2021-05-23

**Authors:** Gianna Maria Nardi, Roberta Grassi, Artnora Ndokaj, Michela Antonioni, Maciej Jedlinski, Gabriele Rumi, Katarzyna Grocholewicz, Irena Dus-Ilnicka, Felice Roberto Grassi, Livia Ottolenghi, Marta Mazur

**Affiliations:** 1 Department of Oral and Maxillo-Facial Sciences, Sapienza University of Rome, Via Caserta 6, 00161 Rome, Italy; giannamaria.nardi@uniroma1.it (G.M.N.); artnora.ndokaj@uniroma1.it (A.N.); antonioni.michela@gmail.com (M.A.); livia.ottolenghi@uniroma1.it (L.O.); 2 Department of Biomedical Sciences, University of Sassari, 07100 Sassari, Italy; grassi.roberta93@gmail.com; 3 Department of Interdisciplinary Dentistry, Pomeranian Medical University in Szczecin, 70-204 Szczecin, Poland; maciej.jedlinski@pum.edu.pl (M.J.); katgro@pum.edu.pl (K.G.); 4 Foundation Policlinico Universitario Agostino Gemelli IRCCS, Largo A. Gemelli 8, 00168 Rome, Italy; gabriele.rumi@policlinicogemelli.it; 5 Department of Oral Pathology, Wroclaw Medical University, ul. Krakowska 26, 52-425 Wrocław, Poland; irena.dus-ilnicka@umed.wroc.pl; 6 Department of Basic Medical Sciences, Neurosciences and Sense Organs, University of Bari Aldo Moro, 70122 Bari, Italy; feliceroberto.grassi@uniba.it

**Keywords:** oral microbiome, early life, one thousand days, pregnancy, vaginal delivery, c-section, newborn, breastfeeding, antibiotics, oral health

## Abstract

(1) Background: The purpose of the study was to comprehensively analyze the relationship between the mother’s oral microbiome, modes of delivery and feeding, and the formation of the newborn child’s oral microbiome. (2) Methods: This systematic review included a search through MEDLINE (PubMed) database (from 2010 to July 2020). Research was registered in PROSPERO under the number CRD42021241044. (3) Results: Of the 571 studies, 11 met the inclusion criteria. Included studies were classified according to (i) child’s delivery mode, (ii) maternal exposure to antibiotics and disinfectants, and (iii) feeding type. (4) Conclusions: The interpretation of these papers shows that the type of delivery, maternal exposure to disinfectants and antibiotics during delivery, maternal health classed as overweight, gestational diabetes mellitus, and feeding type are correlated to changes in the maternal and neonatal early oral microbiomes, based on the analysis provided in this systematic review. Because no evidence exists regarding the impact of maternal diet and maternal oral health on the establishment and development of the early oral newborn microbiome, more studies are needed to deepen the knowledge and understanding of the subject and develop preventive and therapeutic strategies of support to pregnant women.

## 1. Introduction

In recent years, much research has focused on analyzing the establishment and consequent development of the gut microbiome after birth [1]. It is widely recognized that the gut microbiome is derived from the mother, and it is likely to be influenced by multiple perinatal and early-life environmental factors such as pregnancy, gestational age, maternal health, mode of delivery, birth weight, type of and duration feeding [2], exposure to antibiotics before, during, and after delivery, and exposure to pets [3]. It is shown that the early-life gut microbiota influences immunological, endocrine, and neural pathways and plays an important role in infant development. It is also recognized as a major contributor to short- and long-term human health [4,5,6].

Plenty of evidence demonstrates that the establishment of the human gut microbiome begins at birth but continues to develop a succession of taxonomic abundances for 2 to 3 years until it reaches adult-like diversity and proportions [2]. Moreover, it is shown that imbalances in the gut microbiota in early life are associated with specific childhood or adult disease outcomes, such as asthma, atopic dermatitis, diabetes, allergic diseases, obesity, cardiovascular disease (CVD), and neurological disorders [7].

In this clinical scenario, The European Perinatal Health Report, published in 2018 from data of 2015, explored maternal and newborn health and care. The report showed that in Europe, the median Caesarean section (C-section) rate is 27.0%, and C-section birth rates were 4% higher in 2015 than in 2010. One quarter of countries have rates below 21%, but this represents an average including much greater increases in countries such as Romania, up by 27% (from 36.9% to 46.9%), Poland by 24% (from 34.0% to 42.2%), Hungary by 21% (from 32.3% to 39%), and Scotland by 17% (from 27.8% to 32.5%). In addition, the EPHR showed that having children later in life is a general trend in Europe [8].

In addition to the above, obstetric procedures and clinical guidelines concerning antibiotic prophylaxis vary in different countries and even in different hospitals within the same country. The increasing age of pregnant women makes C-section the preferred method in order to lower the risk for both mother and child. Prophylactic antibiotics given to women undergoing C-section are clearly advantageous in the prevention of maternal wound infection, endometritis, and serious infectious complications, but uncertainty exists about the long-term effects on the newborn, making the assessment of overall benefits and harm difficult [9].

Early nutrition—breastfeeding and formula milk—may have long-lasting metabolic impacts into adulthood [10]. For example, it is documented that formula feeding is associated with altered body composition in infancy compared with breastfeeding [11].

Early nutrition impacts bodily systems such as the immune system and the central nervous system during critical temporal windows of perinatal development [2]. The first 1000 days are considered susceptible timepoints for insults that can have long-lasting effects on the microbiota–gut–brain axis [12].

Finally, studies have shown that the oral microbiome is above the gut microbiome, indicating that the fetal gut is colonized early on by bacteria from swallowing in utero and later becomes colonized by bacteria from the placental microbiome. The placental microbiome is very similar to the human oral microbiome and the full-term neonatal microbiome, and thus, the range of oral microbial species that the infant is exposed to orally, and any subsequent selection among those species, determines the establishment of the gut microbiome [13].

The initial exposure to microorganisms is believed to be important as it defines the successive trajectories that lead to a complex and stable ecosystem in adulthood [14]. However, the influence of these factors on the formation of the oral cavity habitat is unknown. Moreover, only scanty data are available on the effect of maternal oral health on the establishment and development of the newborn oral microbiome and the source of the initial neonatal microbiome, and the factors dictating initial human oral microbiota development are unknown.

The purpose of this systematic literature review is to comprehensively and widely analyze the relationship between the mother’s oral microbiome, modes of delivery and feeding, and the formation of the newborn child’s oral microbiome. Multiple and different parameters such as delivery by C-section, characteristics of the newborn, antibiotic use, and mother’s vegetarian or vegan diet are evaluated as co-factors in the evolution of microbiomes.

## 2. Materials and Methods

This systematic review was conducted in accordance with the Preferred Reporting Items for Systematic Reviews and Meta-Analyses (PRISMA) statement and the guidelines from the Cochrane Handbook for Systematic Reviews of Interventions. The study protocol was registered after the screening stage (PROSPERO CRD42021241044).

### 2.1. Eligibility Criteria

The following inclusion criteria were applied for this meta-analysis: (1) randomized clinical trials (RCTs); (2) cohort studies; (3) cross-sectional studies; (4) case-control studies; (5) pilot studies; (6) prospective and observational studies; (7) all considered participants being neonates delivered vaginally or by C-section; (8) type of infant formula; and (9) studies published in English, Italian, French, German, Spanish, Polish, Albanian, and Portuguese. Broad inclusion criteria have been used to ensure the maximum possible sensitivity. The following were the exclusion criteria: (1) in vitro RCTs; (2) lack of effective statistical analysis; and (3) abstract and author debates or editorials.

The outcomes to be assessed are listed as follows: type of delivery, type of infant formula, analyzed microbiome and the technique used, use of antibiotics, number of bacterial CFU: (i) protective against caries as (*Lactobacillus* spp. (LB); (ii) strongly associated with cariogenicity, such as *Streptococcus mutans* (SM); (iii) putative periodontal pathogens such as *Porphyromonas gingivalis* (PG), *Aggregatibacter actinomycementcomitans* (AA), *Prevotella intermedia* (PI).

### 2.2. Search Strategy and Study Selection

Literature searches of free text and MeSH terms were performed using MEDLINE (PubMed) from 2010 to July 2020. All searches were conducted using a combination of subject headings and free-text terms. The final search strategy was determined through several pre-searches. The keywords used in the search strategy were as follows: (((“breast-feeding”)) OR (“breast feeding”)) OR (((((((“pregnant wom*”[All Fields] OR (((((“infant, newborn”[MeSH Terms] OR (“infant”[All Fields] AND “newborn”[All Fields])) OR “newborn infant”[All Fields]) OR “newborn”[All Fields]) OR “newborns”[All Fields]) OR “newborn s”[All Fields])) AND (((“caesarean”[All Fields] OR “caesareans”[All Fields]) OR “cesarean”[All Fields]) OR “cesareans”[All Fields])) OR ((((((((“anti bacterial agents”[Pharmacological Action] OR “anti-bacterial agents”[MeSH Terms]) OR (“anti bacterial”[All Fields] AND “agents”[All Fields])) OR “anti bacterial agents”[All Fields]) OR “antibiotic”[All Fields]) OR “antibiotics”[All Fields]) OR “antibiotic s”[All Fields]) OR “antibiotical”[All Fields]) AND (((“pregnancy”[MeSH Terms] OR “pregnancy”[All Fields]) OR “pregnancies”[All Fields]) OR “pregnancy s”[All Fields]))) OR (((“diet, vegetarian”[MeSH Terms] OR (“diet”[All Fields] AND “vegetarian”[All Fields])) OR “vegetarian diet”[All Fields]) OR (“vegetarian”[All Fields] AND “diet”[All Fields]))) OR “vegan diet”[All Fields]) AND (((((((“microbiome s”[All Fields] OR “microbiomic”[All Fields]) OR “microbiomics”[All Fields]) OR “microbiota”[MeSH Terms]) OR “microbiota”[All Fields]) OR “microbiome”[All Fields]) OR “microbiomes” OR “oral hygiene”).

Reference lists of primary research reports were cross-checked in an attempt to identify additional studies. Following the inclusion criteria, two authors (MM and GR) independently selected the literature by reading the titles and abstracts. The full text of each identified article was then read to determine whether it was suitable for inclusion. Disagreements were resolved through consensus or by discussion with a third author (AN).

### 2.3. Data Collection

For each eligible study, data were independently extracted by two authors (MM and MA) and examined by the third author (AN) by creating a piloted spreadsheet and comparing them through it, in accordance with the Cochrane Collaboration guidelines. In cases of missing data, MM contacted the corresponding author of the related research via email and excluded those ones for which no reply was received.

### 2.4. Data Items

The following data items were recorded: author, study year, study type and setting, age, recruitment sample and its size, site sample, case and control interventions, any pre-treatment and co-intervention, the analyzed microbiome and the technique used, type of delivery, type of infant formula, the influence of delivery mode and body habitat on the neonate’s first microbiota, the intake or not of antibiotics by mothers and neonates, follow-up, drop-out and sample size at follow-up, and strengths and weakness of each study included in the qualitative synthesis.

### 2.5. Quality Assessment

According to the PRISMA statements, the evaluation of the methodological quality provides an indication of the strength of evidence provided by the study, as methodological flaws can result in biases. This procedure provides a total score that can range from 0 to 9, where 0 is a low-quality study, and 9 is the highest possible quality. A trial is considered as having a good quality when it gets a score of at least 5.

### 2.6. Risk of Bias in Individual Studies

Selection bias (retained allocation concealment), performance and detection bias (blinding of participants and operators), attrition bias (patient dropout, missing values or participants, too short duration of follow-up), and reporting bias (selective reporting, unclear eliminations, missing results) were recorded, evaluated, and allocated in accordance with Cochrane guidelines.

## 3. Results

### 3.1. Study Selection

**Figure 1:**
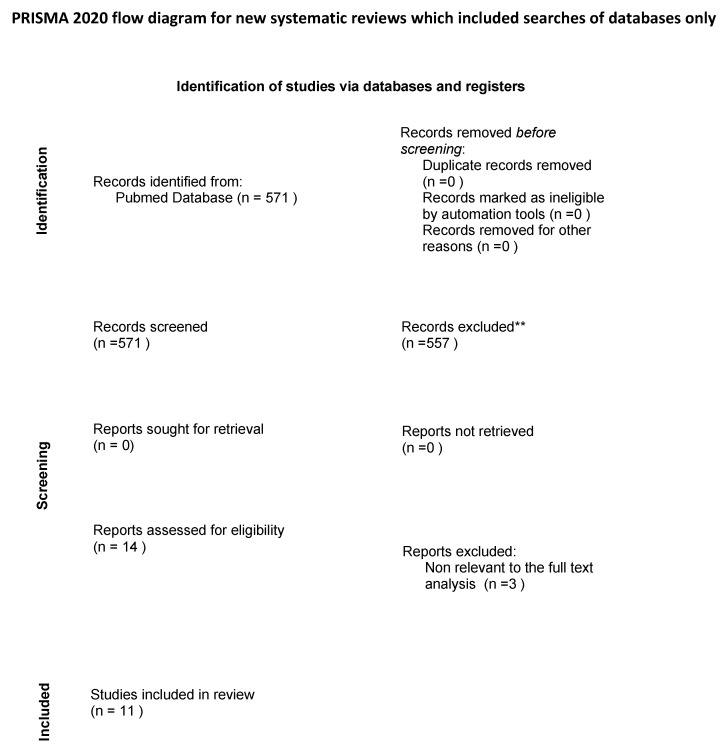
Flow diagram of the search strategy.

The search strategy identified 571 potential articles from PubMed. Of these, 557 papers were excluded because they did not meet the inclusion criteria. Of the remaining 14 papers, 3 were excluded because they were not relevant to the subject of the study. The remaining 11 papers were included in the qualitative synthesis. (Figure 1). Table 1 summarizes the characteristics of each of the 11 included studies [15,16,17,18,19,20,21,22,23,24,25].

### 3.2. Study Characteristics

The included studies (Table 1) were published between 2010 and 2019 and used a cohort design (n = 11). Sample sizes ranged between 19 and 486 participants (neonates and mothers). The overall sample size was 870 (mean = 171). All the included studies presented with oral microbiome analyses in the newborn, immediately after birth, and in a few cases with a prolonged period of follow-up. The oral microbiome was analyzed in mothers (i) before delivery, from the third semester of pregnancy; (ii) immediately after delivery (seconds); and (iii) in a few cases with a period of follow-up (from 6 to 16 weeks after delivery). In addition to oral microbiomes from saliva, mucosa and/or plaque, microbiomes from nares, skin, oropharyngeal, and meconium were also collected in newborns, and from the mouth, nose, skin, vagina, colostrum, and breast milk in pregnant women. During library preparation and microbiome sequencing in the presented studies, Illumina MiSeq platforms, popular and efficient in the process of the microbiome evaluation, were used. The number of results provided in each study required a variety of software to be used for the analysis of the data from the sequencing—some custom-made and some created by the research units.

The included studies were classified according to (i) delivery mode, (ii) maternal exposure to antibiotics and disinfectants, and (iii) feeding type.

#### 3.2.1. Delivery Mode

Delivery mode (C-section or vaginal) was reviewed in 10 of the studies [16,17,18,19,20,21,23,24,25]. Women presented as overweight or obese in one study [22], and in another with gestational diabetes mellitus (GDM) [19].

Delivery mode was associated with vertical transmission of *Lactobacillus* from the mother to the newborn [16,20,21,24], demonstrating that vaginally delivered infants developed oral microbiome communities mirroring their own mother’s vaginal microbiota, characterized by *Lactobacillus*, *Sneathia* spp., and *Prevotella*, while those born by c-section developed bacterial communities similar to those found on the mother’s skin, characterized by *Staphylococcus, Propionibacterium* spp., *and Corynebacterium*.

Chu et al. aimed to assess the impact of delivery mode on taxonomy and metabolome of the neonatal and early infant across several body locations, immediately after birth and at 6 weeks of life. The authors showed that immediately after birth, slight variations in the taxonomy of microbiome in the oral cavity, nares, and skin were associated with c-section, but at 6 weeks of life, no discernible differences in either community structure or function by Caesarean delivery were identifiable. Chu et al. concluded that within the first 6 weeks of life, the offspring microbiome undergoes significant reorganization that is primarily related to body site and not to mode of delivery [18].

Thakur et al. followed 60 mother–infant pairs for 1 year, 30 delivered vaginally and 30 with C-section. The oral swab samples of the newborns were collected for the detection of *S. mutans*. The study results showed that prolonged bottle feeding, socioeconomic status, and tasting of food by the mothers were correlated with early colonization of *S. mutans* in the oral cavity of infants [25].

#### 3.2.2. Exposure to Antibiotics and Disinfectants

Two studies explored the effect on the oral microbiome in the newborn after maternal exposure to antibiotics intrapartum [22,24], while one study focused on the role of intrapartum vaginal disinfection with iodine povidone [20].

Gomez-Arango et al. profiled the placental, oral, and gut microbiomes from 36 overweight or obese mother–baby dyads as determined by 16 S rRNA sequencing. The neonatal oral microbiota was 65.35% of maternal oral, 3.09% of placental, 31.56% of unknown, and 0% of maternal gut origin. In addition, the intrapartum exposition of the mother to antibiotics was correlated to two different neonatal oral microbiome profiles: one strongly similar to the maternal oral and one with less resemblance. Families belonging to *Streptococcaceae, Lactobacillales*, and *Gemellaceae* were dominant in unexposed neonates, while the families belonging to Proteobacteria were predominant after exposure to antibiotics. Moreover, 26% of the exposed newborns presented with the Vim-1 antibiotic resistance gene. These results demonstrated that maternal intrapartum exposure to antibiotics is a key regulator of the initial oral microbiome in the newborn [22].

Keski-Nisula et al. showed that maternal intrapartum antibiotics and prolonged expectant management after rupture of membranes were associated with a decreased transmission rate of vaginal Lactobacillus flora to the neonate during birth [24].

Li et al., in a sample of 20 cases of full-term vaginally delivered neonates (randomly divided into two groups, the conventional disinfection group and the non-disinfection group, with an additional simultaneous 10 infants with elective C-section taken for comparison), showed that the genus *Lactobacillus* presented extremely low levels in the C-section and the disinfection groups, whereas it was the absolute dominant bacterium in the non-disinfection group. Moreover, the authors showed that in the disinfection group, *Prevotella*, *Escherichia–Shigella*, *Staphylococcus,* and *Klebsiella* increased significantly, and through the Kyoto Encyclopedia of Genes and Genomes pathway analysis (KEGG PATHWAY DATABASE), the authors found that there were more harmful pathways, such as Staphylococcus aureus infection, viral myocarditis, and sporulation in the disinfection group [20].

#### 3.2.3. Feeding Type

Three studies focused on feeding type, mainly breast- and formula feeding, and their correlation with the oral microbiome in newborns. Timby et al. characterized the oral microbiota in infants at 4 and 12 months of follow-up fed with milk fat globule membrane-supplemented formula and compared it to that of infants fed standard formula or breast milk. The results showed that infants in the breastfeeding group had significantly lower species richness at 4 months of age, and their microbiota pattern differed markedly from that of the formula-fed groups. Moreover, Moraxella catarrhalis was less prevalent in infants fed milk fat globule membrane-supplemented formula than in those fed standard formula, and the authors hypothesized that it might be associated with the decrease in otitis media seen in the same group [23]. Holgerson et al. compared the oral microbiota in breast-fed and formula-fed infants and investigated growth inhibition of *Streptococci* by infant-isolated *Lactobacilli*. They showed that at 3 months of age, *Lactobacilli* were cultured from 27.8% of exclusively and partially breast-fed infants, but not from formula-fed infants. Furthermore, isolates of *L. plantarum, L. gasseri*, and *L. vaginalis* inhibited the growth of the cariogenic *S. mutans* and the commensal *S. sanguinis: L. plantarum* > *L. gasseri* > *L. vaginalis* [15]. Al-Shehri et al. highlighted the role of breastfeeding with evidence that maternal milk interacts with baby saliva with simultaneous production of reactive oxygen species and growth-promoting nucleotide precursors. Maternal milk has more than a nutritional function in mammals, and interaction with baby saliva generates an effective combination of inhibitory and stimulatory metabolites that appears to determine the early oral—and therefore gut—microbiome composition. Subsequently, milk–saliva mixing acts as a distinct biochemical synergism, which appears to enhance early innate immunity [17].

### 3.3. Quality Assessment

According to the PRISMA statements, the evaluation of methodological quality gives an indication of the strength of evidence provided by the study, which is important because methodological flaws can result in biases [26].

In accordance with the Newcastle–Ottawa scale (NOS) on cohort studies [27], the authors evaluated the qualities of all 11 trials included in the qualitative synthesis, based on object selection, comparability, and outcome. For each item, a series of response options is provided. A star was described as an appropriate entry, with each star representing 1 point. The quality assessment score ranged from 0 to 9 points, with a high score indicating a good-quality study (Table 2).

## 4. Discussion

The purpose of this systematic literature review was to comprehensively and widely analyze the relationship between the mother’s oral microbiome and the formation of the newborn child’s oral microbiome, in relation to modes of delivery, feeding types, and exposure to disinfectants and antibiotics during delivery.

Interestingly, the current systematic literature review could not, on the basis of the included studies, draw conclusions about a possible correlation between: (i) qualitative analysis of the mother’s microbiome in relation to the assessment of the mother’s oral health (presence of caries, periodontitis, inflammatory conditions, prosthetic and implant rehabilitation) and (ii) impact of the maternal oral health on the development of the oral microbiome of the newborn. Thus, this systematic review could not highlight the possible susceptibility of the newborn to the development of clinical conditions that characterize the state of the maternal oral cavity.

On the other side, the health of mothers and newborns are essential indicators of population health and wellbeing. Good health during pregnancy and at birth extends beyond the perinatal period and is an important element for the later health. Perinatal exposures and outcomes during pregnancy are associated with increased predisposition to asthma, metabolic diseases, allergies, obesity, and hypertension. The Euro-Peristat Project emphasizes that the health of the mother is the health of the child and that provision of care and support to the mother during pregnancy must be guaranteed in order to ensure the wellbeing of their newborn babies [8].

Among the prenatal factors, GDM and overweight status of the mother were shown to correlate with maternal and newborn microbiome alterations. The microbiome of the expectant woman can vertically transmit to the newborn. GDM is a disease of abnormal glucose tolerance that first occurs and is recognized during pregnancy. The study by Wang et al. showed that GDM correlated to taxonomic homogeneity across several sample types, and concordant was the variation between mothers and newborns. Metabolic depletion in the newborn gut microbiome was correlated with the microbial shifts, resulting in augmented prevalence of distinct viruses in the meconium. The results of this study show that GDM can modify both the maternal and the offspring microbiome and underline the importance of understanding the formation of the early-life microbiome in the light of the evident maternal microbiome inheritance [19].

The study by Gomez Arango et al. on gut and oral microbiota from 36 obese and overweight mother–newborn dyads showed that the maternal oral, placental, and maternal gut microbiomes were represented by 65.35%, 3.09%, and 0%, respectively, in the newborn oral microbiome. Interestingly, 31.56% of the oral newborn microbiome was of unknown origin. Moreover, maternal exposition to intrapartum antibiotics segregated two different neonatal oral profiles: one strongly resembling the maternal oral microbiome in unexposed newborns and one with less similarity being correlated with antibiotic exposition [22].

Data on intra-uterine microbial colonization have questioned the paradigm that fetal development occurs in a sterile environment. Mothers are the principal source of bacteria for neonates, but it is unclear whether the shaping of the newborn microbiome is the result of mother-to-newborn transmission before, during, or after birth [28]. The evidence of bacterial communities in meconium [29], amniotic fluid [30], placenta [31], and fetal membranes [32], and the fact that the maternal microbiota is needed to shape the offspring’s immune system [33], all indicate a possible host–microbial interaction in utero. Disturbance of microbiota transmission from mother to infant has been connected with obesity [33], type I diabetes [34], asthma [35], and neurodevelopment later in life [36].

The perinatal factors occurring during delivery and after the rupture of membranes that have been described in the present review are mode of delivery, exposure to antibiotics, and use of disinfectants. The findings of these studies strongly reaffirm that vertical transmission of the microbiome occurs from the mother to the newborn, and that a different mode of delivery selects the associated oral microbiome in the newborn. It was shown that vaginally delivered neonates developed bacterial communities that were similar to the vaginal microbiome of the mothers, dominated by *Lactobacillus, Prevotella*, and *Sneathia*, while those delivered by C-section harbored bacterial communities that showed similarity to the mothers’ skin communities, predominantly characterized by *Staphylococcus* and *Corynebacterium* [16].

In this process, the use of intrapartum disinfectants and antibiotics was shown to have a role in selecting the microbiome bacteria. The study by Li et al. on 30 newborn babies, 10 vaginally delivered with disinfection and 10 without disinfection, and 10 delivered by C-section, showed that disinfection with iodine povidone increased the potentially harmful bacteria such as *Prevotella*, *Staphylococcus*, *Klebsiella*, and *Escherichia–Shigella*, in contrast to the C-section and vaginal groups. In the study, the samples of oral swab were collected within 1 min after birth of the head with no contact with the mother’s skin, and Lactobacillus had a high relative abundance only in the non-disinfectant group. The authors concluded that vaginal delivery after disinfection may be more detrimental than C-section delivery to the establishment of infant oral microflora. Moreover, evidence is needed on the effect of disinfection on the mother’s health and postpartum recovery [20]. The disinfectant’s effect on the decrease in the transmission rate of vaginal *Lactobacillus* flora to the neonate during birth was also confirmed by the study of Nisula et al., who investigated the consequences of intrapartum antibiotics [24].

Among the various environmental postnatal factors that the newborn is exposed to, feeding type was shown to play an important role in newborn oral microbiome formation. The results of this systematic review confirm that the oral microbiome differs between bottle- and breastfeeding newborns. The main outcomes were the *Lactobacillus* counts. Holgerson showed that at 3 months of age, *Lactobacilli* were cultured exclusively in the oral swabs from the babies who were breastfed even if this was not the exclusive method of feeding used. Saliva from bottle-fed infants presented no *Lactobacilli* at all. This study is in accordance with previous reports that demonstrated that breast milk provides nutrition for the infant and is a source of *Lactobacilli, Bifidobacteria*, and *Streptococci* [37]. Moreover, early colonization of *Lactobacillus* flora might play a preventive role in the development of allergic diseases later in life. Breast milk components also inhibit growth and attachment of bacteria, such as the caries pathogens *S. mutans* and *Candida Albicans* [38], but they promote attachment of other *Streptococcus* and *Actinomyces* species [15]. Therefore, breast milk is likely to affect the establishment of the microbiota in the mouth as well as in the gut [15]. Finally, the authors suggest that oral flora in breast-fed infants were potentially more associated with long-term effects on early childhood caries, but gut microflora possibly also played a role [15].

The idea of maternal and newborn health and access to care is related to the “First Thousand Days” concept. It refers to the period from conception to the child’s second birthday and is increasingly gaining traction as a concept for guiding public health policy. It is widely recognized as a crucial window of opportunity for interventions that improve child and population health [39]. In recent years, Gasbarrini et al. have concentrated much effort on studying gut microbiota formation, establishment, and possibility of modulation in health and disease. A very recent review by the research group of Prof. Gasbarrini recognizes the results of the present systematic review and in particular states that each human’s gut microbiome is shaped in early life, and this arrangement depends on (i) several perinatal factors such as birth gestational date, type and time of delivery, and type and duration of feeding and weaning and (ii) external factors such as antibiotic use [40].

This is in accordance with our results. The roles of both the mother and of various later environmental factors are identified as participants in the process of establishment and formation of the newborn’s microbiome community. Interestingly, again, no evidence has been found in this work on the role of maternal oral health as a status that can eventually select the microbiome community if a disease is present, such as caries, periodontitis or periimplantitis, and then vertically transmit it to the newborn [40,41].

Mothers kiss their babies—it is a very common behavior found in almost all mammals, including humans. Kissing is shown to activate the immune system, and in particular memory B cells, as well as being an important part of baby–mother bonding [42].

Gasbarrini et al. further highlight that after the first thousand days of their formation, the personal native core microbiome remains relatively stable during adulthood [40]. Evidence exists that enterotypes, body mass index, lifestyle, sport, and cultural and dietary habits are putative factors of the interindividual variation of the core adult microbiome. Certainly, dysbiosis of the gut microbiome is not only associated with intestinal disorders, but also with several extra-intestinal diseases such as neurological and metabolic disorders [40].

In this clinical scenario, the broadest and most comprehensive understanding of the influence of the mother’s oral health on the formation of the newborn’s oral microbiome, and hence on the formation of the early intestinal microbiome, is essential in order to develop innovative and promising preventive and therapeutic interventions for women who are planning pregnancy.

Clinical trials are required to study possible correlations between the oral health of the mother, the maternal oral microbiome, and the establishment and formation of the early newborn oral and gut microbiomes.

## 5. Conclusions

Based on the results of this systematic review, it can be concluded that type of delivery, maternal exposure to disinfectants and antibiotics during delivery, maternal overweight status, GDM, and feeding type are correlated with changes in the maternal and neonatal early oral microbiome. This underlines the direct correlation of the maternal oral and neonatal microbiomes and strengthens the evidence for vertical transmission between them.

Interestingly, no evidence exists on the impact of (i) maternal diet and (ii) qualitative assessment of the maternal oral microbiome correlated to maternal oral health (presence of caries, periodontitis, inflammatory conditions, prosthetic and implant rehabilitation) regarding the establishment and development of the early oral newborn microbiome. Further studies are needed to deepen the knowledge and understanding of these latter factors in order to develop preventive and therapeutic strategies of support to pregnant women.

## Figures and Tables

**Table 1:** Characteristics of each of the 11 included studies.

Study	Analyzed Microbiome	Sample Size	Total Size	Antibiotics/ Disinfection	C-Section Delivery	Vaginal Delivery	Formula Feeding	Breast Feeding	Strengths
Li H (2019)	NEONATES: oral secretions	30 infants	30	povidone iodine	10	20 (10 disinfected by povidone iodine, 10 no disinfection)	-	-	The mode of delivery affects the infant’s *Lactobacillus* level obtained from the mother. Infants with vulvar disinfection presented lower *Lactobacillus* more similar to the C-section than the non-disinfection group, but also more opportunistic pathogens than the C-section group.
Wang J (2018)	NEONATES: saliva, pharyngeal aspirates, meconium, and amniotic fluid PREGNANT WOMEN: saliva, feces and vaginal secretions	140 neonates, 346 women	486	NR	140	-	-	-	The microbial shift in maternal microbiota of different body sites may be associated with GDM.Bacterial abundance between groups at the phylum level was analyzed, with the largest changes seen in the oral cavity (more *Proteobacteria*). Species richness of the pharyngeal and amniotic fluid samples was even comparable to the maternal oral and intestinal communities. The microbial composition and variation of mother and newborn could be driven by the health status of the pregnant woman. The effects of GDM on microbes in pregnancy might be vertically transmitted to the baby during pregnancy.
Li H (2018)	NEONATES: saliva	94 neonates, 94 women	188	NR	18	74 (27 not included because multiple vulvar sterilization during 24 h long delivery)	-	-	The differences in oral microflora between groups can be attributed to vaginal contact and manifest that the microbial environment of babies depends on different modes of delivery. *Lactobacillus, Prevotella*, and *Gardnerella* were the most abundant genera in the vaginal group, while *Petrimonas, Bacteroides, Desulfovibrio, Pseudomonas, Staphylococcus, Tepidmicrobium, VadinCA02,* and *Bifidobacterium* were dominant bacteria in the C-section group.
Chu DM (2017)	NEONATES: nares, skin, oral, meconium PREGNANT WOMEN: nares, skin, oral, stool, introitus, post. fornix	PW(n: 81) at 3rd semester + PW(n:81) recruited at delivery	326	NR	52 (from 157 sampled)	105 (from 157 sampled)	-	-	A significant amount of heterogeneity was seen in the neonatal oral and gut metagenomes at delivery, particularly in the neonatal oral cavity. The mode of delivery was associated with differences in the neonatal microbiota immediately after delivery only within the nares, skin, and oral cavity.
Gomez-Arango LF (2017)	NEONATES: oral swabs PREGNANT WOMEN: placenta, oral swab (16 pairs also having maternal fecal)	36 neonates, 36 overweight mothers	72	Tot = 21; cephazolin (n = 15) and benzylpenicillin(n = 6)	16	20	35	1	The infant’s mouth is colonized by bacteria that resemble those of the mother’s mouth; the first newborn oral bacteria may be maternal in origin, and the oral microenvironment distinctively stimulates colonization by specific bacteria. This study confirms that the effect of intrapartum antibiotics also affects the composition of the oral microbiota in newborns, regardless of the mode of delivery.
Timby N (2017)	NEONATES:At 4, 12 months, buccal mucosa, tongue, alveolar ridges;At 12 m, also teeth;At both 4 and 12 m, saliva	240 neonates	240	NR	205	35	160 (80: experimental formula;80: standard formula)	80	At 12 months, the presence of *S. mutans* is more prevalent in formula-fed than in breast-fed infants. There is different composition of the oral microbiota in the breast-fed compared with the formula-fed infants.
Al-Shehri SS (2015)	NEONATES: saliva WOMEN: colostrum, breastmilk	60 neonates, 77 healthy adults	137	\	0	60	-	60	The interaction of maternal milk with neonate saliva produces peroxide. The composition of oral microbiota of neonates affects their health, as the gut is colonized by microbiomes originating from the mouth. The neonatal salivary pattern develops into the adult pattern over a period between 6 weeks and 6 months. This early and unpredicted transition confirms a correlation between neonatal oral microbiome and weaning.
Keski-Nisula L (2013)	NEONATES: oralWOMEN: vaginal fluid, oral	45 neonates, 45 women	90	Antibiotics to the mother during the intrapartum period before birth after rupture of membranes: 17	4	41			Maternal intrapartum antibiotics and prolonged expectant management after ROM were associated with decreased vertical transmission rate of vaginal *Lactobacillus* flora to the neonate. As early colonization of *Lactobacillus* flora may have a preventive role in the development of allergic diseases later, the significance of intrapartum prophylactic antibiotics needs to be highlighted in forthcoming studies, especially as regards immunological development of the offspring.
Holgerson PL (2013)	NEONATES: mucosa of the cheeks, tongue, and alveolar ridges	169 neonates (207 infants (3 m old)	169	NR	41	166	23	146, + 38 partially breastfed,	The observed differences in the gastrointestinal tract microbiota composition due to feeding mode extend to the oral cavity, and viable *Lactobacilli* detected in saliva from breastfed, but not formula-fed, infants had an inhibitory effect on oral *Streptococci*. *Lactobacilli* isolated from oral, breast milk, and other non-oral sites inhibit growth of selected oral pathogens, especially cariogenic *Streptococcus mutans* and *Candida albicans*.
Thakur R (2012)	NEONATES: oral saliva or plaque sample, dorsum of tongue (before the eruption of teeth) or from the alveolar ridge and teeth (after the eruption)	60 neonates, 60 women	120	NR	30	30			Saliva is a reflection of overall oral flora and study specimen in the predentate. Breast feeding plays a preventive role in colonization of *S. mutans*. Human breast milk contains inhibiting factors (immunoglobulins, antibodies, etc.) specific for particular genotypes of *S. mutans* harbored in a child’s mouth. Prolonged bottle feeding with bovine milk and sucrose results in intermittent pooling of milk on the tooth surface. This appears to be associated with early establishment of *S. mutans* in the oral cavity.
Dominguez-Bello MG (2010)	NEONATES: nasopharynx, oral mucosa, and skin PREGNANT WOMEN: skin, oral mucosa, vagina	10 neonates, 9 women (46 body sites in newborns, 34 body sites in women)	19	1 (7th month of pregnancy)	5	4			Infants born vaginally acquire bacterial communities resembling their own mother’s vaginal microbiome, dominated by *Lactobacillus, Sneathia* spp., and *Prevotella*, while those born by c-section develop bacterial communities similar to those found on the mother’s skin, characterized by *Staphylococcus*, *Propionibacterium* spp., and *Corynebacterium*.

**Table 2:** Newcastle–Ottawa quality assessment scale for cohort studies.

NEWCASTLE-OTTAWA QUALITY ASSESSMENT SCALE–COHORT STUDIES												
Author		Dominguez-Bello 2010	Holgerson 2013	Al-Shehri 2013	Keski-Nisula 2013	Derrick 2017	Wang 2018	Li 2019	Li 2018	Gomez-Arango 2017	Timby 2017	Thakur 2012
**Selection** (Maximum 4 stars)	(1) Representativeness of the exposed cohort	*	*	*	*	*	*	*	*	*	*	*
	(2) Selection of the non-exposed cohort	*	*	*	*	*	*	*	*		*	*
	(3) Ascertainment of exposure	*	*	*	*	*	*	*	*	*	*	*
	(4) Demonstration that outcome of interest was not present at start of study			*		*				*	*	
**Comparability** (Maximum 2 stars)	(5) Comparability of cohorts on the basis of the design or analysis **	*	**	*	*	**	*	*	*	**	*	*
**Outcome** (Maximum 3 stars)	(6) Assessment of outcome	*	*	*	*	*	*	*	*	*	*	*
	(7) Was follow-up long enough for outcomes to occur?					*	*				*	*
	(8) Adequacy of follow-up of cohorts					*					*	*
	**Total score**	5	6	6	5	9	6	5	5	6	8	7

A study can be awarded a maximum of one star *. for each numbered item within the Selection and Outcome categories. A maximum of two stars ** can be given for Comparability.

## Data Availability

Not applicable.
